# Predictors of Emergency Room Access and Not Urgent Emergency Room Access by the Frail Older Adults

**DOI:** 10.3389/fpubh.2021.721634

**Published:** 2021-09-03

**Authors:** Susanna Gentili, Leonardo Emberti Gialloreti, Fabio Riccardi, Paola Scarcella, Giuseppe Liotta

**Affiliations:** Department of Biomedicine and Prevention, University of Rome “Tor Vergata,”Rome, Italy

**Keywords:** frail older adults, emergency department, functional geriatric evaluation, emergency room utilization, social determinants, health determinants

## Abstract

**Background:** Emergency rooms (ERs) overcrowded by older adults have been the focus of public health policies during the recent COVID-19 outbreak too. This phenomenon needed a change in the nursing care of older frail people. Health policies have tried to mitigate the frequent use of ER by implementing community care to meet the care demands of older adults. The present study aimed to investigate the predictors of emergency room access (ERA) and not-urgent emergency room access (NUERA) of community-dwelling frail older adults in order to provide an indication for out-of-hospital care services.

**Method:** Secondary analysis of an observational longitudinal cohort study was carried out. The cohort consisted of 1,246 community-dwelling frail older adults (over 65 years) in the Latium region in Italy. The ER admission rate was assessed over 3 years from the administration of the functional geriatric evaluation (FGE) questionnaire. The ordinal regression model was used to identify the predictors of ERA and NUERA. Moreover, the ERA and NUERA rate per 100 observations/year was analyzed.

**Results:** The mean age was 73.6 (SD ± 7.1) years, and 53.4% were women. NUERAs were the 39.2% of the ERAs; robust and pre-frail individuals (79.3% of the sample) generated more than two-third of ERAs (68.17%), even if frails and very frails showed the higher ER rates per observation/year. The ordinal logistic regression model highlighted a predictive role on ERAs of comorbidity (*OR* = 1.13, *p* < 0.001) and frailty level (*OR* = 1.29; *p* < 0.001). Concerning NUERAs, social network (*OR* 0.54, *P* = 0.015) and a medium score of pulmo-cardio-vascular function (*OR* 1.50, *P* = 0.006) were the predictors.

**Conclusion:** Comorbidity, lack of social support, and functional limitations increase both ERA and NUERA rates generated by the older adult population. Overall, bio-psycho-social frailty represents an indicator of the frequency of ERAs. However, to reduce the number of ERAs, intervention should focus mainly on the robust and pre-frail needs for prevention and care.

## Introduction

During the twenty-first century, public health policies have constantly focused on overcrowding of the Emergency Departments (EDs) by older adults (1–3). However, some authors have shown how public policy needs to reduce overcrowding to guarantee satisfactory care quality and safety (4, 5). The Australasian College for Emergency Medicine had defined Emergency Room (ER) overcrowding as “the percentage of patients who were admitted or planned for admission but discharged from the ED without reaching an inpatient bed, transferred to another hospital for admission, or died in the ED whose total ED time exceeded 8 h” (6, 7).

Several studies have focused on the significant characteristics of an ER user to define the “frequent user” (2, 8–11). However, there was no unique definition of the frequent use that could include patients who access the ED from 2 to 12 times per year (2, 8). Despite the complexity of the phenomenon, Wang et al. (12) have identified the shortage of ED beds available compared to the high number of patients accessing the ER daily as the leading cause of overcrowding. Moreover, Erenler et al. (13), have analyzed the impact on the overcrowding of the frequent users, highlighting the need to manage the repetitive admissions. Given the multidimensional nature of the overcrowding phenomenon, a single cause has not been highlighted. The most significant reasons seem to be the inappropriate use of EDs (14, 15) and the lack of “long-term care” (2, 16), specifically those which are aimed at frail older people. Other authors have focused on the significant consequences of overcrowding (12, 17, 18). The big factors associated with overcrowding seem to increase adverse outcomes for the patient and worsen the quality of care (12, 17, 18).

The consequences of this phenomenon gain even more relevance as a result of the recent COVID-19 outbreak. This pandemic demanded a rapid health system reorganization (19–21) because of the crucial role of EDs (21). A systematic review by Aminzadeh et al. (22) drew attention to the inappropriate use of EDs by older adults and the complex clinical characteristics of this population due to the high presence of comorbidities. Other studies emphasized the complexity of the frail elderly care needs, increasing the risk of readmissions after discharge (2, 23, 24).

Moreover, some authors have investigated the importance of social support on Emergency Room Access (ERA) of older adults, even if in a systematic review by Valtorta et al. (25), there was no significant association between the ERA of older adults and the social support. Nevertheless, lack of social support and disability seems to be the strongest independent determinants for increasing the occurrence of adverse outcomes among older adults (26) and the use of hospital services, such as ERA, hospital admissions, and Day Hospital services. However, the analysis of the determinants of ED accesses has not dedicated sufficient consideration to the aspect of multidimensional frailty, defined as a dynamic state determined by the loss of one or more functional areas (clinical physical, cognitive, psychological, functional, social, and economic) which causes a higher increase in the risk of adverse outcomes as mortality and hospitalization (27).

The purpose of this study is to investigate the predictors of ERA and Non-Urgent Emergency Room Access (NUERA) by community-dwelling frail older adults.

## Methods

### Study Design

This is a secondary analysis of an observational longitudinal cohort study whose main aim is to assess frailty in community-dwelling older people. Recruitments started in January 2014 and finished in December 2017. A detailed description of the survey (28) and follow-up (29, 30) has been published elsewhere.

### Participants

The sample was enrolled in 2014 from a population aged over 64 years resident in the Lazio region (Italy). After the recruitment and the assessment of frailty, the sample was followed up for 3 years. Eligibility criteria for baseline recruitment were: (a) age of 65 years or higher; (b) residence in the Lazio region, except for those living in an institution; (c) people with cognitive impairments were included in the study thanks to the support of caregivers. According to the inclusion criteria, 1,331 individuals aged more than 64 years participated in the study. During the 3-year follow-up, 84 people were lost mainly because of residence change, so the sample involved in this study consists of 1,247 individuals.

### Data Collection

At baseline, block randomization was performed to represent the Lazio region resident population aged over 64 years. Initially, a randomization list was drawn from the local health authorities (LHA) archives in order to select a group of general practitioners (GPs) to be involved in the study. Subsequently, randomization was performed by sampling from the GPs list to place a maximum of 25 patients over 64 years. The aims of the study were explained to GPs and patients, and then, all the participants signed the informed consent form.

After 3 years, follow-up data collection was conducted upon administrative data of admissions recorded by the regional health database. The regional health database collects all health services provided by the regional hospitals.

### Outcome

The primary outcome of the study was to explore the association between the level of frailty, disability, and comorbidity, and ERA and NUERA.

The outcome variables analyzed in this study were:

- ERA: the absolute frequency of ERA for each participant, along with the assessment of the level of frailty during the 3-years of follow-up. Moreover, the ERA rate per 100 observations/year has been analyzed.
- NUERA: the frequencies of NUERA, defined as all the ERAs classified as “non-critical state of health; immediate care is not required” by the triage personnel.

### Measurement

The functional geriatric evaluation (FGE) questionnaire (31) was administered to assess the multidimensional bio-psycho-social frailty. FGE stems from the Grauer functional rating scale (32), modified and validated for the Italian population by Palombi et al. (31, 33, 34). This questionnaire stratifies the population according to the level of frailty (robust, pre-frail, frail, and very frail) associated with a growing risk of mortality, hospitalization, and institutionalization (28, 30, 35). FGE collects sociodemographic data and information on five domains: physical health, mental health, functional state, social resources, and economic resources. These domains contributed to the final score (FS), ranging from −108 to 101. According to FS, the final synthetic score (FSS) identified the level of frailty as: very frail (score ≤10), frail (score >10 but <50), pre-frail (score ≥50 but ≤70), robust (score >70). With the support of the GPs, the presence or absence of the disease was ascertained for each participant.

To define disability, Activities of Daily Life (ADL) and Instrumental Activities of Daily Life (IADL) were assessed (36, 37). Moderate disability corresponded to any dependence in performing IADL and severe disability to any dependence in performing ADL. The absolute number of ERAs as well as the urgency code to identify NUERA has been retrieved from the Regional Health Database. The frequent ERA users were defined as elderly with two or more access per year.

### Ethical Consideration

All the data collection was performed in line with the ethical standards of the 1965 Declaration of Helsinki and subsequent amendments. The Independent Ethical Committee of the University of Rome "Tor Vergata” approved the study (registration number: 95/15). Written consent was obtained by all the participants before data collection.

### Statistical Analysis

The statistical analyses were carried out with IBM SPSS Statistics version 25.0. The absolute number of ERA and NUERA rates have been calculated for each person, and the NUERA and ERA rate per 100 observations/year is stratified for frailty level. The one-way ANOVA analysis was accomplished to compare the mean rates. Descriptive statistics, such as means, SD, frequencies, and percentages, were used to describe the sociodemographic characteristics of the sample. Univariate and bivariate analyses (Spearman's correlations or chi-square) have been performed to select the variables (the ones analyzed by the FGE questionnaire plus ADL and IADL, Table 1 and Supplementary Tables 2–4) associated with the dichotomized ERA and NUERA (no access vs. any access). Moreover, the descriptive statistics and univariate analyses were performed to address the 84 individuals lost during the follow-up compared to the total sample (Supplementary Table 1). A chi-square on contingency tables was carried out to select the variables included in the multivariate model, and statistical significance was determined by a value of *p* < 0.05. Finally, the variables that showed a statistically significant association with the ERA and NUERA were included as covariates in a final multivariable generalized linear (GENLIN) ordinal regression model (38). The ordinal regression analysis was appropriate because the dependent variables (NUERA and ERA) were included as ordinal variables (no access, 1, 2, 3, and >3 accesses). The use of the SPSS GENLIN model aimed to explore which covariate was independently associated with NUERA and ERA. The fit model was assessed with Akaike Information Criteria (AIC) and Bayesian Information Criteria (BIC) measures.

**Table 1:** Sociodemographic characteristics of the sample (*N* = 1,247).

		**No-ERA**	**ERA**	**tot**.	**χ^2^*p*-value**
		***N* (%)**	***N* (%)**	***N* (%)**	
Age	<74	384 (47.5)	159 (36.3)	543 (43.5)	0.001
	75–85	339 (41.9)	221 (50.5)	560 (44.9)	
	>86	86 (10.6)	58 (13.2)	144 (11.6)	
Gender	Female	450 (55.6)	215 (49.1)	665 (53.4)	0.027
Education	No education	57 (7.1)	39 (8.9)	96 (7.7)	NS
	Primary school	379 (46.8)	211 (48.2)	590 (47.4)	
	Middle school	203 (25.1)	107 (24.4)	310 (24.8)	
	High school	129 (16.0)	60 (13.7)	189 (15.2)	
	Degree	40 (5.0)	21 (4.8)	61 (4.9)	
Cohabitants	Alone	162 (19.8)	98 (22.4)	260 (20.7)	NS
	Spouse	422 (52.2)	223 (50.9)	645 (51.7)	
	Child	180 (22.2)	91 (20.8)	271 (21.7)	
	Others	28 (3.5)	14 (3.2)	59 (3.4)	
	Home worker	19 (2.3)	12 (2.7)	31 (2.5)	
Frailty	Robust	382 (47.2)	160 (36.6)	542 (43.5)	
	Pre-frail	282 (34.9)	157 (35.8)	439 (35.2)	<0.001
	Frail	99 (12.2)	75 (17.1)	174 (14.0)	
	Very frail	46 (7.9)	46 (10.5)	92 (7.3)	
Comorbidity	Yes	768 (94.9)	425 (97.0)	1,193 (95.7)	<0.001
Disability	No	599 (74.0)	267 (61.0)	866 (69.5)	<0.001
	Moderate	170 (21.1)	126 (28.7)	296 (23.7)	
	Severe	40 (4.9)	45 (10.3)	85 (6.8)	

*No-ERA, no emergency room access; ERA, emergency room access, χ^2^, chi-square of the cross table; NS, not statistically significant. No-ERA was for the sample which did not access the ER during the 3 years of follow-up; ERA was for those who had one or more access to the ER during the follow-up*.

## Results

Of the 1,331 eligible patients at baseline, 1,247 (93.68%) were included in 2017 during follow-up. The sociodemographic and clinical characteristics of the final sample are shown in Tables 1, 2.

**Table 2:** Prevalence of ERA (*N* = 823) and NUERA (*N* = 323), and one-way ANOVA of ERA and NUERA rate (per 100 observations/year), stratifies for frailty levels.

	ERA^**^		NUERA^**^	
	***N* (%)**	**Rate** **(per 100 observation/ year)**	***N* (%)**	**Rate** **(per 100 observation/ year)**
Robust	263 (31.96)	20.89	115 (35.60)	8.16
Pre-frail	298 (36.21)	32.70	109 (33.75)	11.15
Frail	171 (20.77)	68.33	76 (23.53)	22.43
Very frail	91 (11.06)	64.55	23 (7.12)	23.01

*ERA, emergency room access; NUERA, non-urgent emergency room access;*

** *-value of one-way ANOVA < 0.001*.

Patients are mostly women (53.4%) and, the sample average age is 73.64 (SD ±7.16). Patients belong mainly to two age groups, <74 years and between 74 and 85 years old, 43.5% and 44.9%, respectively. The education achievement level is more represented by those who have left at primary school (47.4%) than those who have a higher educational level (middle school 24.8% and high school 15.2%), and most of them live with their spouse (51.7%), their children (21.7%), or alone (20.7%).

Based on the FSS, the sample was 43.5% robust, 35.2% pre-frail, 14.0% frail, and 7.3% very frail.

**Figure 1:**
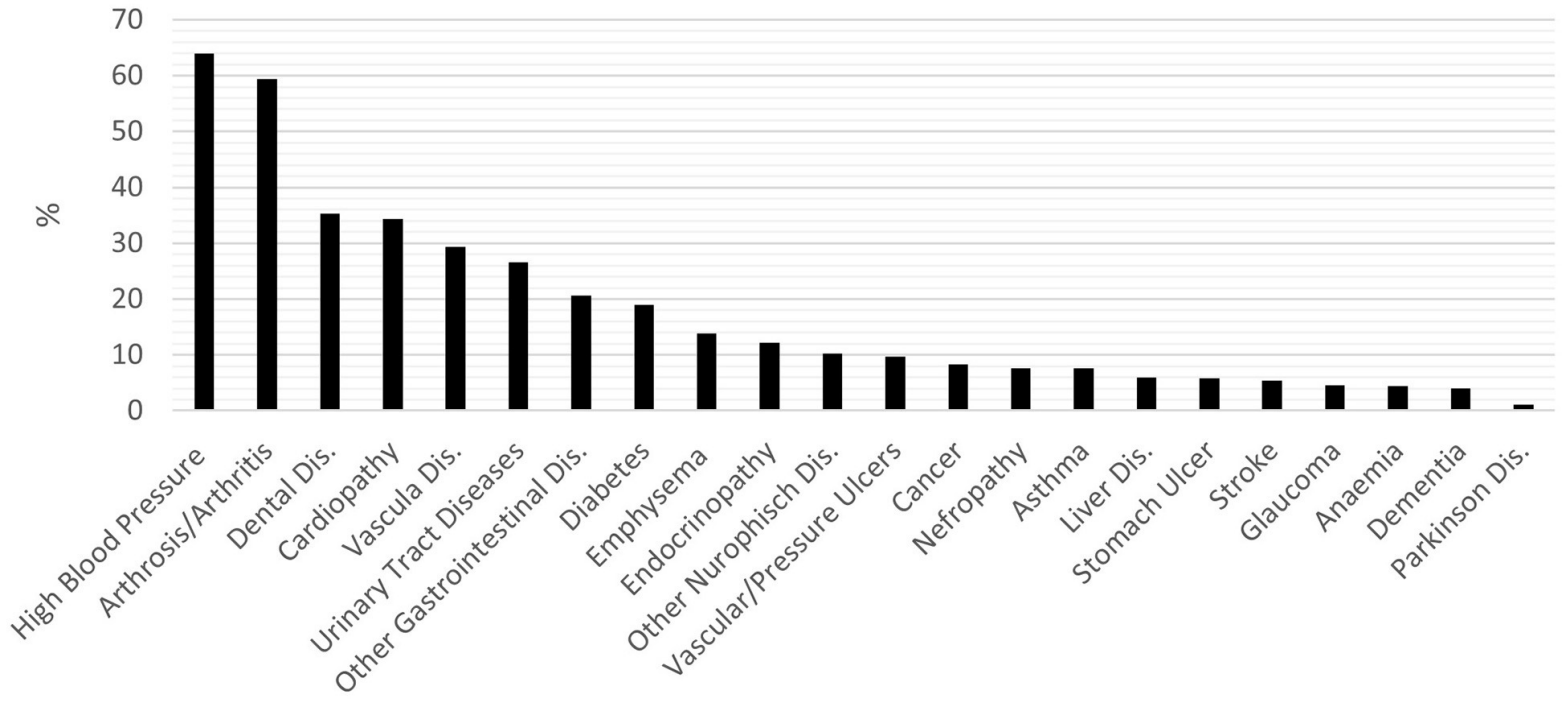
Prevalence of disease among the sample.

The cumulative percentage of individuals with comorbidities (two or more active diseases) was 95.7%. Figure 1 shows the prevalence of the disease in the sample. The most frequent pathologies are cardiovascular (hypertension, cardiopathy, vascular diseases, and vascular or pressure ulcers, 63.99, 34.32, 29.35, and 9.62%, respectively), arthrosis or arthritis (59.34%), dental diseases (35.28%), and urinary tract diseases (26.62%).

Sociodemographic variables of the 84 individuals who were lost during follow-up (see Supplementary Table 1) did not differ from the total sample except for gender.

The specific characteristics of the population significantly associated (*P* < 0.05) with more access to the ER (Table 1) are: being men (50.9%), age between 75 and 85 years (50.5%), and comorbidity (97.0%) but with moderate disability condition (28.7%). Overall, to be frail or pre-frail was associated with higher number of ERA (35.8 and 36.6, respectively).

During 3 years of the study, 35.1% of the sample (438 individuals) accessed the ER department at least once and generated 823 accesses, of which 39.24% were NUERA (Table 2). The frequent ERA users were 6.1% (elderly with two or more access per year). The ERA and NUERA rates were 34.89 per 100 observations/year [95% *CI* 29.06; 40.71] and 12.30 [95% *CI* 9.84; 14.76], respectively. The one-way ANOVA shows (Table 2) a significant difference according to the level of frailty, both for ERA rate *F*_(3,1243)_ = 11.94, *p* < 0.001, and for NUERA rate *F*_(3,1243)_ = 6.61, *p* < 0.001.

The percentage of ERA and NUERA stratified by level of frailty is shown in Table 2. The total number of ERAs carried out by the sample was 823, of which 31.96% were made by robust, 36.2% by pre-frail, 20.8% by frail, and 11.1% by very frail. For NUERA, the total accesses were 323 of which 35.60, 33.75, 23.53, and 7.12% were carried out by robust, pre-frail, frail, and very frail, respectively.

A univariate analysis was conducted before choosing the variable to insert in the predicting model (as shown in Supplementary Tables 1–3). In the Supplementary Tables, the single items of the FGE (as shown in Supplementary Table 2), the prevalence of disease (as shown in Supplementary Table 3), and ADL/IADL (as shown in Supplementary Table 4) were analyzed to evaluate the level of correlation with the outcome variable, ERA, and NUERA. Although some variables were significant on univariate analysis, they did not explain the dependent variable when introduced in the multivariate model.

The ordinal logistic regression (GENLIN) model was carried out to identify the predictors of ERA and NUERA (Table 3). Patients had significantly more ERAs if they were men (*OR* 1.54, *P* < 0.001, 95% *CI*: 1.22; 1.95). The risk of high number of ERAs increased with increased frailty levels (*OR* 1.29, *P* < 0.001, 95% *CI* [1.13; 1.47]). Finally, the person with comorbidities had a significantly increased risk of ERA than their counterparts (*OR* 1.13, *P* < 0.001, 95% *CI* [1.06; 1.20]).

**Table 3:** Ordinal logistic (GENLIN) models were predicting determinants of ERA and NUERA.

			**Hypothesis test**				**95% confidence interval**	
**Predictors of ERA**	**β**	**Std. Er**.	**Wald χ^2^**	***df***	***p*-value**	**OR**	**Lower**	**Upper**
Gender (male)	0.434	0.1198	13.126	1	<0.001	1.544	1.221	1.952
Frailty	0.256	0.0663	14.844	1	<0.001	1.291	1.134	1.47
Comorbidity	0.123	0.0313	15.356	1	<0.001	1.131	1.063	1.202
**Predictors of NUERA**								
Social network	−0.607	0.2498	5.901	1	0.015	0.545	0.334	0.889
Pulmo-Cardio-Vascular Function (Medium score)	–.410	0.1484	7.620	1	0.006	1.506	1.126	2.015
Pulmo-Cardio-Vascular Function (Bad score)	−0.053	0.3479	0.023	1	0.880	0.949	0.480	1.876

*ERA, emergency room access. Dependent variables: absolute number of ERA and NUERA. Covariate's variable = Comorbidity and frailty. Test omnibus χ^2^_(3)_ =51.73, P < 0.001; ERA AIC = 662.89 and BIC = 698.77. NUERA, not-urgent emergency room access. Dichotomic variable = Social network. NUERA test omnibus χ^2^_(3)_ = 14.049, P = 0.003; NUERA Akaike information criteria (AIC) = 93.75 and Bayesian information criteria (BIC) = 124.52*.

Community-dwelling older people had significantly more NUERA if they had no social network than if they had (*OR* 0.54, *P* = 0.015, 95% *CI* [0.33; 0.89]). Moreover, a medium score of pulmo-cardio-vascular function (“able to climb a flight of stairs or walking for one city block” compared with “no restriction”) increase the number of NUERA (*OR* 1.50, *p* = 0.006, 95% *CI* [1.12; 2.01]) that was not the case for people with more severe limitation compared with “no restriction.”

## Discussion

Frequent access to the ERs has increasingly become a worldwide public health issue with significant consequences (i.e., overcrowding) on the management of EDs (4). This framework is crucial to understand the factors associated with routine access to the ER generated by older adults in order to optimize the resources. This paper aimed to analyze the predictors of ERA and NUERA for community-dwelling older people; moreover, the study addressed to investigate how older adults access the EDs. The main difference between the two models seems to be linked to the level of urgency of the accesses. Accesses associated with a high level of urgency were significantly associated with physical issues, while non-urgent accesses were generated also by social issues. These results can explain predictors involved in the ERAs.

Although there was no clear definition of frequent ED access (10, 39), we used the percentage of frequent users to compare with other international studies on ERA and NUERA rates. Our results show that the frequent ERA users were 6.1% (elderly with two or more access per year). This result agreed with data reported in other studies (8, 9, 40, 41).

Some studies (42–45), both in the United States and Europe, investigated the prevalence of frailty in the ER patient that ranged from 7 to 80%, according to the frailty definition used by the authors. Our study defined frail 21.3% of the population, using a bio-psycho-social description (27). The higher level of frailty has been associated with a higher ED access rate per observation year in the present study as well as in others (43). However, the highest portion of ERA and NUERA is generated by robust and pre-frail patients because of the prevalence of robust and pre-frail older people in the sample (about 80%) and in agreement with other authors (2, 43). Moreover, NUERA represents about 40% of the ERAs independently from frailty, showing that reasons for accessing the ER should be further investigated since only clinical emergencies seem not to explain all the ERAs.

As reported by other authors and confirmed in the current study results, the ER frequent user profile was male (50.9%, *P* = 0.027) (9), aged between 75 and 85 years (44.9%, *P* = 0.001) (46), with comorbidities, namely, with a high prevalence of cardiovascular and urinary tract disease (47).

There is a general agreement to the significant role played by comorbidity on healthcare needs, especially on the ERA for the older adults (48). The findings of the current study demonstrated that a high level of comorbidity was a predictor of a high ERA rate. The management of comorbidities and clinical problems seems to be the primary cause that oriented the EDs to the medical model (48). However, this model did not take into consideration the complexity of this type of patient.

All world countries are dealing with the increase of inappropriate use of ER by the elderly, which results in the EDs overcrowding. We have observed that the pre-frail and robust represented the groups generating the majority of ERA and NUERA. Few studies focused on the NUERA (15, 46). There was confusion on the definition of not critical ERA because it was often associated with the medical point of view (49, 50). The increase of older adults admitted in the ERs with not urgent triage (50) reflects a social need or an inadequate social network to match the needs of individuals for care (51–53). In agreement with these studies, the current study results show that enough social networks decrease the risk of NUERA (*OR* 0.54). Moreover, an important fact has emerged from the current research: 39.24% of the total ERA was NUERA, confirming the international trends (50, 52, 54). Faulkner et al. argue that a directly proportional link between the increase in the elderly population and the inappropriate use of EDs could influence ER overcrowding (50). A 2013 mixed-method study revealed that a critical cause of increasing the number of non-urgent accesses was represented by a long waiting list that prevents access to primary care for the elderly, mainly due to the lack of a well-established primary care system (55). Other authors confirm that these barriers involve a “rational choice” of the patient in accessing the ER rather than primary care (53, 56). The second factor associated with the increase of NUERA is a moderate impairment of cardiovascular and respiratory function related to a generic initial imbalance of physical performance, a sign of not-stabilized clinical issues.

We observed the primary role of robust and pre-frail community-dwelling people in using the ER. Moreover, this study highlighted the need to change the management of older adults at the community level to reduce ER overcrowding, according to the definition of Australasian College for Emergency Medicine (6). The change should address precisely the robust and pre-frail older adults, which account for about 78% of both ERA and NUERA, through community health and social care that stabilize the clinical situation and support socially isolated individuals. Frequently a not-stabilized clinical condition is associated with the lack of social network (i.e., difficulties in following complicated drug schedule that results in reduced adherence to medication prescription or respecting follow-up appointments because of problems in moving alone out of the house).

The present study has some points of strength. First, to our knowledge, this is the only study that examines the association between frail older adults and access to the ER, focusing on not urgent ERA. Moreover, this research is original because the people involved in the study represent the regional population stratified for frailty. According to the bio-psycho-social model, the evaluation of frailty can help an early identification of robust, pre-frail, frail, and very frail people to address an adequate response to prevent an adverse outcome (mortality, hospitalization, and institutionalization or access to EDs).

Finally, the main limitations identified in this study are represented by two key points. The first one is related to the questionnaire of FGE. While this questionnaire is validated and has a higher predictive power of the adverse outcome, it is not widely used. The second is represented by the health service characteristics of the Lazio region; it is characterized by low community services, especially for robust and pre-frail older people. Another limitation linked to the current results showed that a different distribution by gender in the 84 individuals lost during the follow-up compared with the total sample. However, the higher prevalence of men among the lost to follow-up could only strengthen the result that the male gender represents a risk factor for the occurrence of ERA. These features can reduce the international generalizability and reproducibility of the results of the study. Moreover, further studies should address the differences between urgent and not-urgent ERA, focusing on the association with the mix of clinical instability and lack of social network.

## Conclusion

In conclusion, this study has some important implications for public health policy and clinical practice. A paradigm shift is required to lessen the impact of the growing increase in not urgent or inappropriate access by the elderly to the ER. The change should go beyond the clinical model toward a biopsychosocial model by implementing primary care to identify the needs of robust and pre-frail elderly. Early identification can decrease the overcrowding of ERs and improving care for moderate to severe acute cases. Furthermore, primary care should focus on the social support required by these patients. In the future, it will be crucial to conduct more multicenter studies to assess non-urgent access for the frail community-dwelling older population.

## Data Availability Statement

The raw data supporting the conclusions of this article will be made available by the authors, without undue reservation.

## Ethics Statement

The studies involving human participants were reviewed and approved by Independent Ethical Committee of the University of Rome Tor Vergata. The patients/participants provided their written informed consent to participate in this study.

## Author Contributions

GL, PS, and SG: conceptualization. SG, PS, GL, and FR: methodology and writing, reviewing, and editing. SG and LE: data analysis. GL and PS: investigation. PS, GL, and FR: resources. SG, GL, and LE: data curation and visualization. SG, GL, and FR: writing—original draft preparation. PS, GL, and FR: supervision. GL: project administration. All authors have read and agreed to the published version of the manuscript.

## Conflict of Interest

The authors declare that the research was conducted in the absence of any commercial or financial relationships that could be construed as a potential conflict of interest.

## Publisher's Note

All claims expressed in this article are solely those of the authors and do not necessarily represent those of their affiliated organizations, or those of the publisher, the editors and the reviewers. Any product that may be evaluated in this article, or claim that may be made by its manufacturer, is not guaranteed or endorsed by the publisher.
