# Supplementary material for: Predictors of Emergency Room Access and Not Urgent Emergency Room Access by the Frail Older Adults
Source: Front Public Health. 2021 Sep 3;9:721634. doi: 10.3389/fpubh.2021.721634 (PMC8446358; doi:10.3389/fpubh.2021.721634)
Supplement: Supplementary file 1 [file Table_1.DOCX]

Supplementary Material

# Supplementary Figures and Tables

Supplementary Table 1. Sociodemographic variables of who were lost during follow-up (N=84) compared with the total sample (N=1247)

|  | Follow-Up Lost | Total Sample | P-value |
| --- | --- | --- | --- |
| Gender  Male  Female | 29 (34.5)  55 (65.5) | 665 (53.3)  582 (46.7) | 0.001 |
| Age (mean ± S.D.)  <74  75-85  >86 | 76.33 ± 7.32  35 (41.7)  40 (47.6)  9 (10.7) | 76.34 ± 7.15  543 (43.5)  560 (44.9)  144 (11.6) | N.S. |
| Frailty (mean ± S.D.)  Robust  Pre-Frail  Frail  Very Frail | 53.77 ± 35.72  34 (40.5)  28 (33.3)  14 (16.7)  8 (9.5) | 58.53 ± 29.26  542 (43.5)  439 (35.2)  174 (14.0)  92 (7.3) | <0.001 |
| Comorbidity (mean ± S.D.)  Yes | 3.05 ± 2.37  60 (71.4) | 3.43 ±2.09  1193 (95.7) | 0.009 |
| Disability  No  Moderate  Severe | 50 (59.5)  27 (32.1)  7 (8.3) | 866 (69.4)  296 (23.7)  85 (6.8) | N.S. |

Supplementary Table 2. Chi-Square of Pearson and ordinal regression between ERA and NUERA, and FGE variables.

|  |  | **ERA** | | | **NUERA** | | |
| --- | --- | --- | --- | --- | --- | --- | --- |
|  |  | **Chi-square of Pearson** | | **Ordinal Regression** | **Chi-square of Pearson** | | **Ordinal Regression** |
| **Variables** |  | **χ² (DF)** | **P-Value** | **OR** | **χ² (DF)** | **P-Value** | **OR** |
| Physical Condition | |  |  |  |  |  |  |
| Eyesight | | 7.87 (8) | .448 |  | 3.79(2) | .150 |  |
| Hearing | | 7,95 (8) | .438 |  | 1.22(2) | .543 |  |
| **Mobility** | | 36,59 (8) | **<.001** | .973 | 5.30(2) | .071 |  |
| **Pulmo-Cardio-Vascular Function** | | 47,52 (8) | **<.001** | .983 | 7.11 (2) | **.028** | .966 |
| **Diet** | | 20,83 (4) | **<.001** | **.861 **** | 4.18 (1) | **.041** | **.914*** |
| Mental Condition | |  |  |  |  |  |  |
| **Disorietation** | | 33.48 (8) | **<.001** | .964 | 1.86(2) | .393 |  |
| **Delusions** | | 18.97 (8) | **.015** | 1.018 | 0.12(2) | .939 |  |
| **Memory Loss** | | 19.38 (8) | **.013** | 1.013 | 1.40 (2) | .497 |  |
| **Energy & Drive** | | 24.93 (4) | **<.001** | **.899*** | 1.42 (1) | .233 |  |
| **Judgment** | | 13.80 (4) | **.008** | .998 | 0.02 (1) | .872 |  |
| **Hallucination** | | 17.16 (4) | **.002** | .952 | 0.32 (1) | .571 |  |
| Functional Abilities | |  |  |  |  |  |  |
| Reads and write letters | | 8.73 (4) | .068 |  | 0.22 (1) | .638 |  |
| Able to use telephone | | 7.07 (4) | .132 |  | 0.25 (1) | .611 |  |
| **Able to bank and shop** | | 12.42 (4) | **.014** | 1.155 | 0.20 (1) | .655 |  |
| **Able to prepare simple meals** | | 15.04 (4) | **.005** | 1.151 | 0.25 (1) | .614 |  |
| **Washes, dresses and toilets self** | | 13.98 (4) | **.007** | 1.007 | 0.27 (1) | .603 |  |
| **Use public transportation** | | 12,26 (4) | **.016** | 1.189 | 0.12 (1) | .725 |  |
| **Take medicine and follow diet** | | 17.64 (4) | **.001** | 1.602 | 0.01 (1) | .903 |  |
| Support from the community | |  |  |  |  |  |  |
| **Social Network** | | 21.09 (4) | **<.001** | **1.818 *** | 8.39 (1) | **.004** | **.380*** |
| **Social Support** | | 17.78 (4) | **.001** | 1.375 | 4.57 (1) | **<.001** | **.380*** |
| Can do the shopping | | 5.67 (4) | .225 |  | 0.62 (1) | .803 |  |
| Available supportive and recreational facilities | | 22.80 (20) | .289 |  | 9.59 (5) | .088 |  |
| **Geographic availability of Services** | | 31.10 (24) | .151 |  | 12.66 (6) | **.049** |  |
| Living quarters | | 4.21 (4) | .378 |  | 0.70 (1) | .401 |  |
| Relatives and friends | | 9.06 (12) | .697 |  | 1.19 (1) | .275 |  |
| Financial Situation | | 13.34 (8) | .101 |  | 0.33 (1) | .562 |  |

Note. ERA, Emergency Room Access; NUERA, Non-Urgent Emergency Room Access; *P-value <0.05; ** P-value<0.001. All the items score of Functional Geriatric Evaluation have been related with the categorical variables of ERA and NUERA, the significant ones (P-value < 0.05) have been included in the predictive model.

Supplementary Table 3. Chi-Square of Pearson between ERA and NUERA, and disease

|  | **ERA** | | **NUERA** | |
| --- | --- | --- | --- | --- |
|  | **Chi-square of Pearson** | | **Chi-square of Pearson** | |
|  | **χ² (DF)** | **P-Value** | **χ² (DF)** | **P-Value** |
| Parkinson Dis. | 5.96 (4) | .202 | 0.20(1) | .651 |
| Dementia | 7.74 (4) | .102 | 0.84 (1) | .358 |
| Anaemia | 3.71 (4) | .446 | 0.29 (1) | .587 |
| Glaucoma | 6.84 (4) | .144 | 0.16 (1) | .687 |
| Stroke | 2.83 (4) | .586 | 2.29 (1) | .130 |
| Stomach Ulcer | 5.89 (4) | .207 | 1.05 (1) | .305 |
| Liver Dis. | 2.019 (4) | .732 | 0.35 (1) | .554 |
| Asthma | 9.06(4) | .060 | 0.73(1) | .391 |
| Nefropathy | 8.69 (4) | .069 | 3.57 (1) | .059 |
| **Cancer** | 10.21 (4) | **.037** | 0.80 (1) | .369 |
| Vascular/Pressure Ulcers | 7.54 (4) | .110 | 0.31 (1) | .573 |
| Other Nurophisch Dis. | 8.17 (4) | .085 | 0.10 (1) | .752 |
| Endocrinopathy | 2.66 (4) | .615 | 3.02 (1) | .082 |
| Emphysema | 6.557 (4) | .161 | 1.14 (1) | .285 |
| Diabetes | 3.27 (4) | .513 | 1.16 (1) | .281 |
| **Other Gastrointestinal Dis.** | 11.38 (4) | **.023** | 4.70 (1) | **.030** |
| **Urinary Tract Diseases** | 17.24 (4) | **.002** | 6.74 (1) | **.009** |
| Vascula Dis. | 9.65 (4) | .047 | 0.31 (1) | .573 |
| **Cardiopathy** | 30.81(4) | **<.001** | 2.62(1) | .105 |
| Dental Dis. | 8.46 (4) | .076 | 1.57 (1) | .210 |
| Arthrosis/Arthritis | 1.79 (4) | .773 | 0.28 (1) | .593 |
| High Blood Pressure | 5.36 (4) | .252 | 0.30(1) | .582 |

Note. ERA, Emergency Room Access; NUERA, Non-Urgent Emergency Room Access.

Supplementary Table 4. Chi-Square of Pearson and Ordinal Regression between ERA and NUERA, and Limitation of ADL and IADL

|  |  | **ERA** | | | **NUERA** | |  |
| --- | --- | --- | --- | --- | --- | --- | --- |
|  |  | **Chi-square of Pearson** | | **Ordinal Regression** | **Chi-square of Pearson** | | |
|  |  | **χ² (DF)** | **P-Value** | **OR** | **χ² (DF)** | **P-Value** | |
| Limitation of ADL | Self feeding | 13.28 (8) | .102 |  | 0.51 (2) | .772 | |
|  | **Dressing** | 27.80 (8) | **.001** | .804 | 1.49 (2) | .473 | |
|  | **Toilet Hygiene** | 34.10 (8) | **<.001** | 1.180 | 2.09 (2) | .351 | |
|  | **Functional mobility** | 40.27 (8) | **<.001** | 1.115 | 2.74 (2) | .253 | |
|  | **In and out of bed** | 34.04 (8) | **<.001** | .833 | 1.49 (2) | .474 | |
|  | **Bathing and showering** | 28.25 (8) | **<.001** | 1.022 | 0.76 (2) | .684 | |
|  | **Continence** | 31.57 (8) | **<.001** | **.666*** | 4.11 (2) | .128 | |
| Limitation of IADL | **Using the telephone** | 26.60 (8) | **.001** | .944 | 1.56 (2) | **.**458 | |
|  | **Mooving within community** | 25.89 (8) | **.001** | .792 | 0.72 (2) | .965 | |
|  | **Shopping for groceries and alimentation** | 19.76 (8) | **.011** | 1.383 | 1.58 (2) | .453 | |
|  | **Preparing meals** | 16.70 (8) | **.033** | 1.012 | 0.73 (2) | .693 | |
|  | **Diy/Washing** | 22.55 (8) | **.004** | .883 | 1.72 (2) | .422 | |
|  | **Taking prescribed medicine** | 27.64 (8) | **.001** | .751 | 12.67 (2) | .233 | |
|  | **Maneaging money** | 30,14 (8) | **<.001** | .984 | 12.49 (2) | .052 | |

Note. ERA, Emergency Room Access; NUERA, Non-Urgent Emergency Room Access; *P-value <0.05. All the items score of ADL and IADL have been related with the categorical variables of ERA and NUERA, the significant ones (P-value < 0.05) have been included in the predictive model.
